# Visualizing interface-specific chemical bonds in adhesive bonding of carbon fiber structural composites using soft X-ray microscopy

**DOI:** 10.1038/s41598-022-20233-4

**Published:** 2022-09-29

**Authors:** Hiroyuki Yamane, Masaki Oura, Noriko Yamazaki, Tomoko Ishihara, Koichi Hasegawa, Tetsuya Ishikawa, Kiyoka Takagi, Takaki Hatsui

**Affiliations:** 1 RIKEN SPring-8 Center, RIKEN, Kouto, Sayo, Hyogo 679-5148 Japan; 2 Chemical Research Department, Research and Innovation Center, Mitsubishi Heavy Industries, Ltd., Minato-mirai, Yokohama, Kanagawa 220-8401 Japan; 3 Manufacturing Technology Research Department, Research and Innovation Center, Mitsubishi Heavy Industries, Ltd., Oye, Nagoya, Aichi 455-8515 Japan; 4 Fixed Wing Aircraft Engineering Department, Integrated Defense and Space Systems, Mitsubishi Heavy Industries, Ltd., Toyoyama, Nishikasugai, Aichi 480-0293 Japan; 5 Present Address: Photon Science Innovation Center, NanoTerasu, Aramaki-aza-Aoba, Sendai, 980-0845 Japan; 6 Present Address: Protein Crystal Analysis Division, JASRI, Kouto, Sayo, Hyogo 679-5148 Japan

**Keywords:** Polymer characterization, Composites, Chemical physics

## Abstract

Adhesion is a technology for assembling carbon fiber (CF) reinforced polymer (CFRP), enabling them to maintain their lightweight and high-stiffness properties. Despite the importance of adhesion, the lack of a molecular-level understanding of the adhesion mechanisms has limited the reliability of adhesion for use in next-generation aircraft and automobiles. Here, we focused on the chemical-state distribution at a practical adhesive interface composed of an epoxy-based adhesive film bonded to an epoxy-based CF matrix. By fluorinating the OH group, we succeeded in visualizing the chemical state at the CF-matrix/adhesive interface using soft X-ray microscopy. The soft X-ray images exhibited a decrease in OH-related signals at the interface due to the local chemical interaction at the epoxy-epoxy adhesive interface. We also found that the N and O Kα signals were observable at the CF's surface, indicating the presence of nitrogen- and oxygen-containing functional groups. Based on these observations, we discuss the molecular-level adhesion mechanism at the CF-matrix/adhesive interface.

## Introduction

Carbon fiber (CF)-reinforced polymers (CFRP) are composite materials. Due to their lightweight and high-stiffness properties, they have recently been utilized in the manufacturing of aircraft and automobiles^1–3^. This use of CFRP helps us move toward a low carbon future by reducing fuel consumption and resultant CO_2_ emission. When assembling CFRPs, a bolted joint undermines the advantages of CFRPs because, for example, hundreds of thousands of bolts and their holes reduce both the lightweight and high-stiffness characteristics^2,3^. Furthermore, arc welding does not work for CFRP-CFRP joining structures^2,3^. Compared to these conventional joining methods, the adhesion technique allows a lighter structure and improves the stress distribution in the joining structure. As the bolted joint and arc welding techniques can be managed by controlling torque and electrical power, the adhesion technique must be controlled by specific bonding factors. In the case of the adhesive joint, one of the most critical parameters is the bond enthalpy, where covalent bonds are stronger than other interactions such as hydrogen bonds and van der Waals forces^4–6^. However, examining the chemical-state distribution at adhesive interfaces in terms of bond strength is challenging. This leads to a lack of molecular-level understanding to control the adhesion strength, resulting in the insufficient reliability of CFRP adhesion technology for manufacturing aircraft and automobiles.

Vibrational spectroscopies^7^, X-ray photoelectron spectroscopy (XPS)^8^, and X-ray absorption spectroscopy (XAS)^9^ have been used to investigate the chemical state of materials. Because the spatial resolution of these conventional spectroscopies is in the (sub-)mm order, the chemical analysis of the sub-μm region is impossible and has been approximated using spatially-averaged chemical information. Microprobe chemical analyses have been required to investigate physical and chemical properties in practical applications^10–12^. Synchrotron X-ray analyses have enabled us to extend the elucidation of geometric, electronic, and chemical states from ideal model systems to practical applications^12^. The use of focused soft X-rays allows the local-spot chemical analysis of materials, including light elements and transition metals^13,14^. Because of the various detection methods, including electrons, transmitted X-rays, and fluorescent X-rays, soft X-ray microscopes (SXMs) provide a versatile technique with fewer restrictions on the sample and its surrounding atmosphere^13–20^.

Using the fluorescence-yield SXM, we reported the physical and chemical states at the model adhesive interface between a polyetheretherketone (PEEK) adherend and a diglycidylether of bisphenol A (DGEBA) adhesive with a 4,4'-diaminodiphenyl sulfone (DDS) curing agent^21^. The DGEBA-DDS/PEEK interface exhibited the multiscale phenomena induced by the plasma pretreatment on the PEEK surface, including the sub-mm complex interface structure, the sub-μm distribution of the functional groups, and interfacial covalent-bond formation. From the microprobe spectroscopic measurements, the interfacial covalent bond was ascribed to esterification between the plasma-induced carboxy (COOH) group on the PEEK surface and the reactive hydroxy (OH) and/or remanent epoxy groups in DGEBA-DDS^21^. This finding revealed the molecular-level origins of the adhesion as well as its unexpected failure in the plasma treatment.

**Figure 1:**
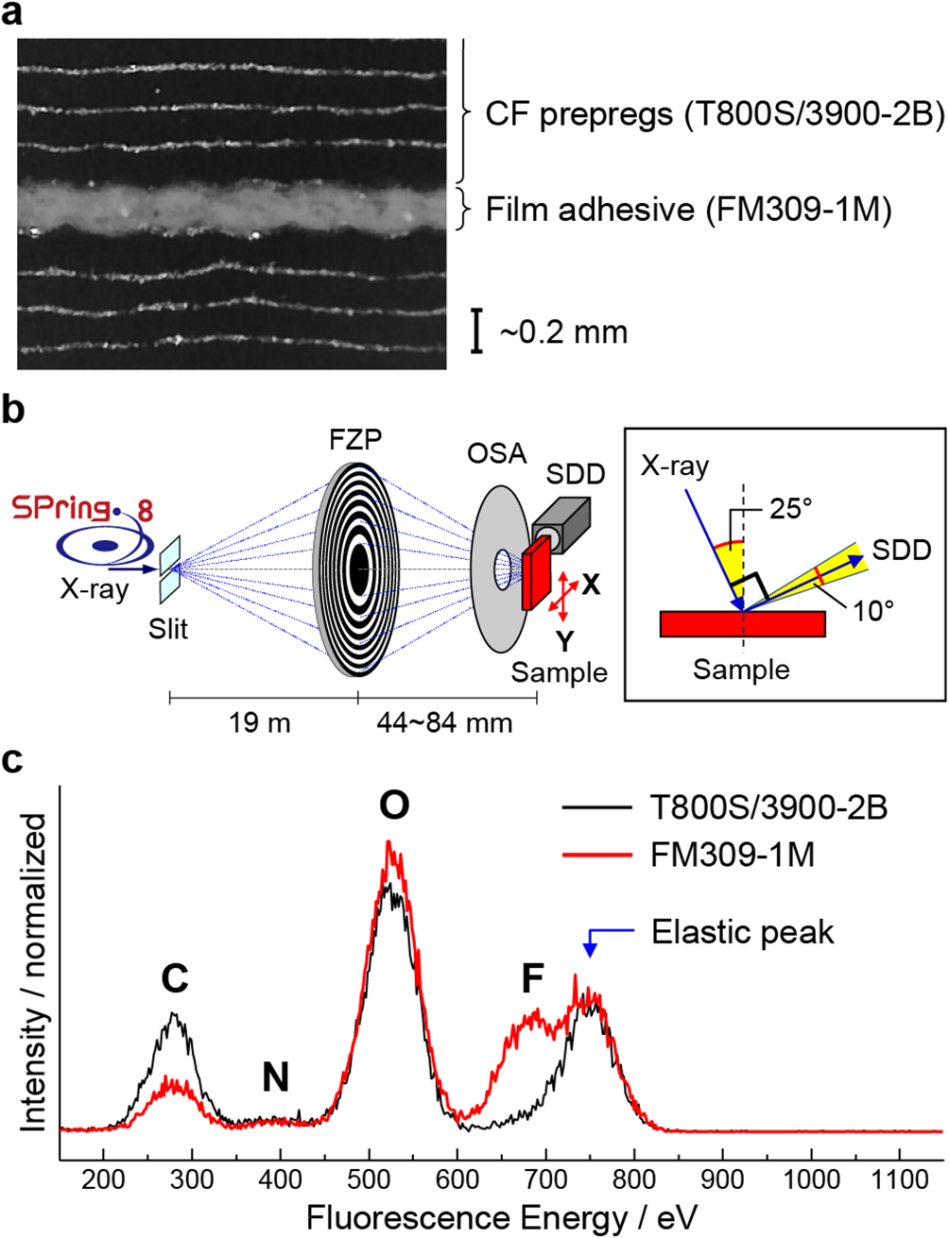
Sample structure, experimental setup, and elemental composition of materials. (**a**) An optical microscope image of the cross-section in the CF prepreg (T800S/3900-2B) joining structure adhered by the adhesive film (FM309-1 M). (**b**) Schematics of the X-ray focusing optics and the measurement geometry of SXM. A soft X-ray beam was focused on using FZP and OSA. *μ*-XRF spectra were measured using SDD with an acceptance angle of 10°. (**c**) *μ*-XRF spectra (*hv* = 750 eV) in the CF region of T800S/3900-2B and the resin region in FM309-1 M. The SXM image was obtained by measuring *μ*-XRF while scanning the sample in the X and Y directions.

In the present work, we discuss the chemical state and its spatial distribution at a practical epoxy/epoxy adhesive interface, which only requires heat treatment without plasma pretreatment for adhesion. As shown in Fig. 1, we examined an adhesive interface between an epoxy-based CF prepreg (T800S/3900-2B, Toray Industries, Inc.)^22,23^ and an epoxy-based adhesive film (FM309-1M, Solvay S.A.)^24^. The CF prepreg (T800S/3900-2B) is constituted of the CF layer and the interlaminar toughened layer in which polyamide particles are dispersed^22^, and the predominant resin in the CF prepreg is a tetra-glycidyl-4,4'-diaminodiphenylmethane (TGDDM) in combination with the DDS curing agent^23^. On the other hand, the adhesive film (FM309-1 M) consists of phenolic epoxy resin and aniline derivative^25^. From the viewpoint of the chemical interactions at the epoxy-based CF-matrix/adhesive interface, we focused on the OH group and the remanent epoxy group, which can form a hydrogen bond and a covalent bond, of thermosetting polymers used in the CF matrix and adhesive. In general, the functional group imaging using SXM depends on the corresponding XAS peak intensity. Because the OH group is composed only of a single bond, corresponding XAS spectra give the broad spectral feature^9,26^, making it difficult to obtain clear image contrast in SXM. Therefore, we fluorinated the OH group with trifluoroacetic anhydride (TFAA)^27–29^, which enabled us to detect the OH-related signal by measuring F Kα X-rays (Fig. 1c). The C, N, O, and F (i.e., OH) Kα imaging based on the microprobe X-ray fluorescence (*μ*-XRF) enabled us to characterize the physical and chemical states at the CF-matrix/adhesive interface. We observed a decrease in the F Kα signal at the CF-matrix/adhesive interface. The decrease in the F Kα (OH) signal at the interface was consistent with the possible formation of covalent bonds between the CF matrix and the adhesive film. Furthermore, using microprobe XAS (*μ*-XAS), we found the presence of oxygen-containing functional groups inside and outside of the CF, which might be introduced to improve the wettability of the CF with its matrix. We discuss the bonding mechanisms at the CF-matrix/adhesive interface based on these observations.

## Results

**Figure 2:**
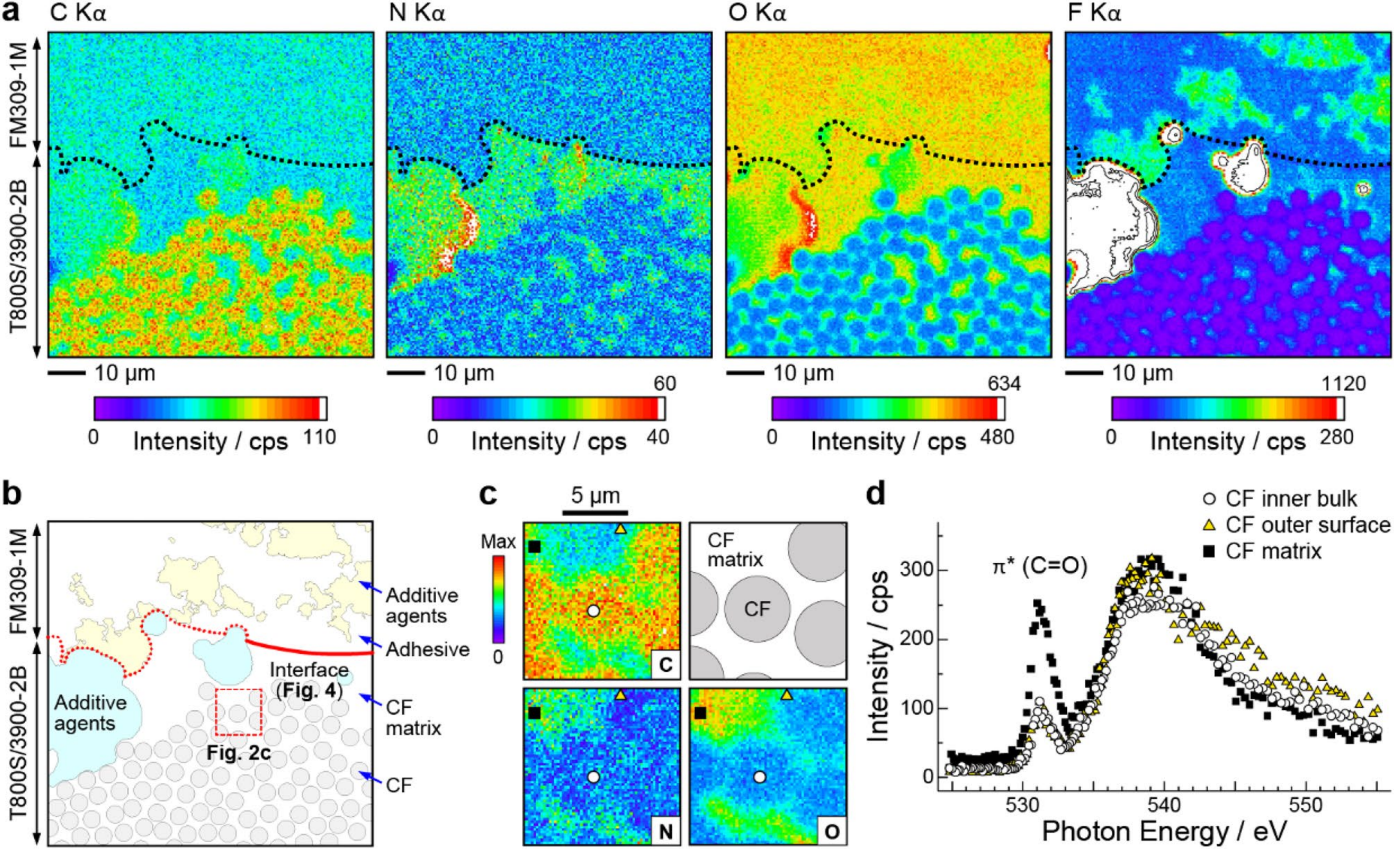
Soft X-ray images of the CF-matrix/adhesive interface. (**a**) The C, N, O, and F Kα SXM images of the CF-matrix/adhesive interface region measured at *hv* = 750 eV. The pixel size was 500 nm square and the dwell time was 1 s/pixel. The dashed curves in the images indicate the interface position, which was determined from the N Kα contrast. Contour lines with a step of 210 counts/s (cps) are shown in the whiteout areas of the F Kα image. (**b**) The segmented image of the CF-matrix/adhesive interface based on the SXM observations. The *μ*-XAS measurement was performed at the dashed square region (**c**). The intensity profile analysis cut across the interface was performed at the thick solid curve region (see Fig. 4a). (**c**) The C, N, and O Kα SXM images with a pixel size of 200 nm square. The circle, triangle, and square symbols in the image indicate the measurement spots of the *μ*-XAS spectra. (**d**) The O K-edge *μ*-XAS spectra measured at the CF inner bulk (circles), the CF outer surface (triangles), and the CF matrix (squares).

Figure 2a shows the C, N, O, and F Kα SXM images measured for the CF-matrix/adhesive interface. The segmented map of the SXM image is also shown in Fig. 2b. The upper and lower regions in the SXM image correspond to the regions of the adhesive (FM309-1 M) and the CF with its matrix (T800S/3900-2B), respectively. We added a dashed line in each image to identify the CF-matrix/adhesive interface position, which was determined from the N Kα contrast. Because both the adhesive and the CF matrix consist of epoxy polymers and some additive agents^22–25^, the SXM images exhibited an element-dependent chemical contrast. The shape of the interface was not straight but irregular at the μm scale, which might be due to the adhesion process (e.g., peel ply just before the adhesion), resulting in mechanical interlocking. These observations indicated the coexistence of both chemical and physical bonding factors in the CF-matrix/adhesive interface.

To understand the chemical interaction at the CF-matrix/adhesive interface, we discuss the SXM image for each element. In the lower half of the C Kα image, multiple circular contrasts with a diameter of about 5 μm were observed. These circles originated from the CF cross-section and agreed well with the catalog diameter of CF in T800S/3900-2B (5 µm)^22^. The interface contrast between the CF matrix and the adhesive was resolved not in the C Kα image but in the N Kα image, where the N Kα intensity of the CF matrix exhibited a stronger intensity than that of the adhesive. The N Kα signal indicated the presence of amine compounds as curing agents for epoxy polymers. The N Kα intensity of the CF matrix, which was about twice as high as that of the adhesive, can be explained by the relative amount of epoxy (i.e., cross-linking) units cured with amine compounds. In the O Kα image, the interface contrast was not resolved, and a mottled chemical contrast was observable in the CF matrix and the adhesive. This mottled chemical contrast was emphasized in the F Kα image, indicating that the mottled contrast could be ascribed to the presence of additive agents with the NH and/or OH groups, which the TFAA treatment could have fluorinated^27–29^. The NH and OH groups in additive agents at the interface region might have introduced the hydrogen-bond interaction.

Based on the SXM images, we discuss the chemical states in the regions of the CF/CF-matrix interface and the CF-matrix/adhesive interface. Figure 2c,d show the C, N, and O Kα images and the O K-edge *μ*-XAS in the CF/CF-matrix interface region. The soft X-ray images of CF exhibited a strong C Kα emission and weak N and O Kα emissions. It has been understood that CF consists of stacks of graphite^30^, which have a hydrophobic character^31^. Observation of the N and O Kα signals from CF suggests that CF's nitrogen- and oxygen-containing functional groups might exist. To confirm the chemical states of CF, the O K-edge *μ*-XAS spectra were measured for the CF inner bulk (indicated by a circle), the CF outer surface (triangle), and the CF matrix (square). For all three positions, the *μ*-XAS spectra exhibited a pre-edge peak at *hv* = 531 eV, originating from the 1 s → π^*^ transition at the C=O site^26^. The chemical images and *μ*-XAS spectra indicated that CF consisted of polar groups including N–H and C=O, which might improve the wettability with the CF matrix. Note that the pre-edge peak at the CF-matrix region was stronger than that at the CF inner bulk and the CF outer surface. This difference can be explained by the presence of the R-O-(C=O)-CF_3_ group in the CF matrix, which was substituted from the R-OH group of epoxy polymers by the TFAA treatment as follows:

$${\text{R - OH }} + \, \left( {{\text{CF}}_{{3}} {\text{CO}}} \right)_{{2}} {\text{O }} \to {\text{ R - O}}\left( {{\text{C}} = {\text{O}}} \right){\text{CF}}_{{3}} + {\text{ CF}}_{{3}} {\text{COOH}}.$$

**Figure 3:**
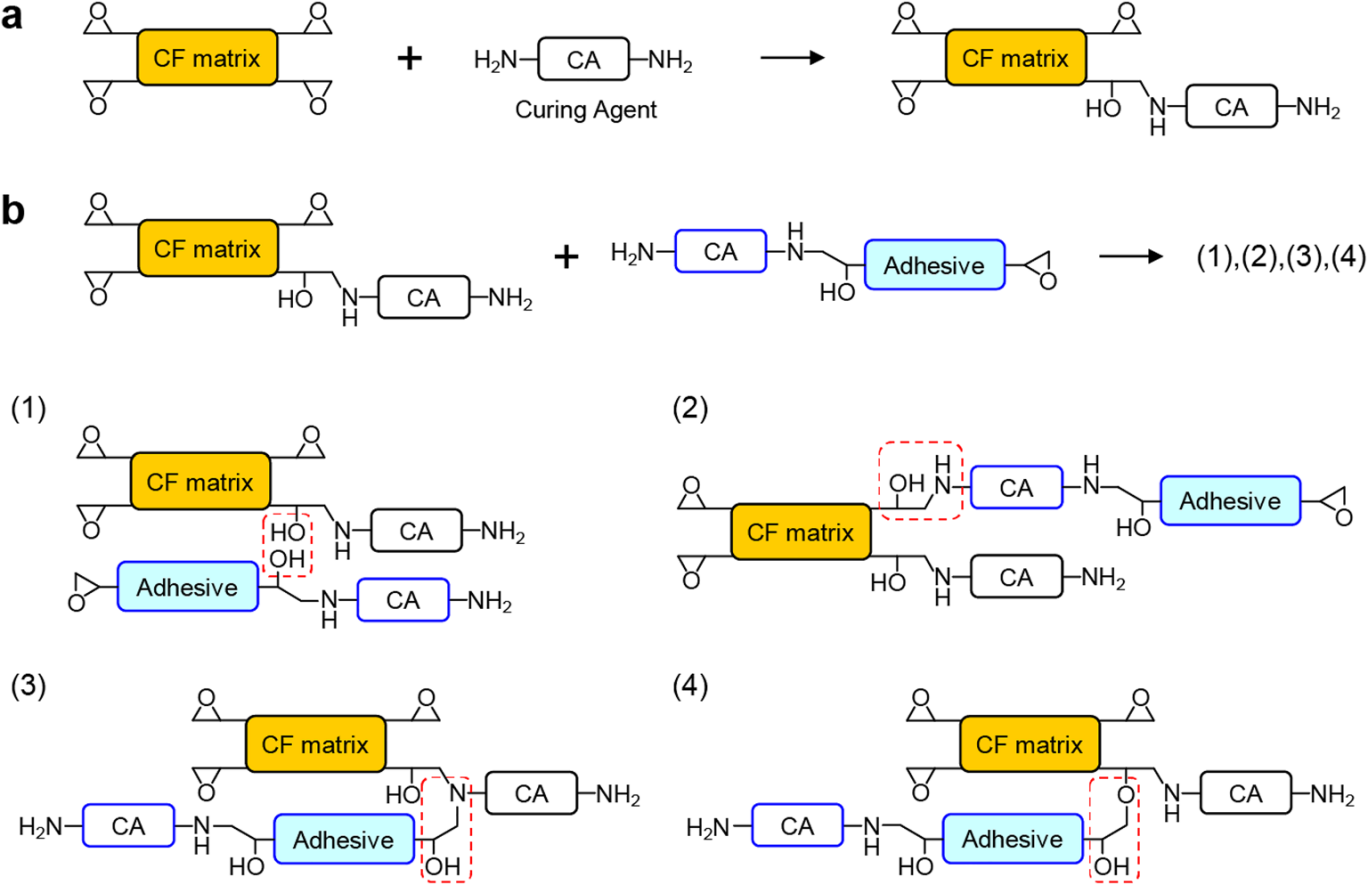
Chemical interactions between the CF matrix and the adhesive. (**a**) Schematics of the curing reaction between the epoxy-based CF matrix and the amine-based curing agent (CA). (**b**) Possible bonding models between the CF prepreg (T800S/3900-2B) and the adhesive film (FM309-1 M), where the reaction site is indicated by the dashed squares. (1) Hydrogen bond between the CF matrix and the adhesive film. (2) Curing reaction of the remanent epoxy group in the CF matrix with the primary amine in the adhesive film. (3) Curing reaction of the remanent epoxy group in the adhesive film with the secondary amine in the CF matrix. (4) Etherification of the remanent epoxy group in the adhesive film with the OH group in the CF matrix.

Next, we discuss the CF-matrix/adhesive interface. Figure 3 shows the possible chemical interactions between the CF matrix and the adhesive in epoxy-amine systems. According to Ref. 23, the predominant resin in the CF prepreg was TGDDM-DDS. As shown in Fig. 3a, the CF matrix (TGDDM) consists of four epoxy groups to form the cross-linking structure with the curing agent (DDS). In this system, four bonding models can be considered at the CF-matrix/adhesive interface, as shown in Fig. 3b. Based on the relative N Kα intensity between the CF matrix and the adhesive in Fig. 2a, we assumed that the number of reactive epoxy groups in the adhesive would be half of that in the CF matrix. If this was the case, a potential epoxy resin would be DGEBA, a well-known thermosetting polymer consisting of two epoxy groups^33^. The first possible bonding, model (**1**), is the hydrogen bond between the OH groups in the CF matrix and that in the adhesive film. Other bonding models (**2**)–(**4**) involve the formation of covalent bonds. Model (**2**) is the ring-opening reaction of the remanent epoxy group in the CF matrix that can react with the primary amine in the adhesive film. Model (**3**) is the ring-opening reaction of the remanent epoxy group in the adhesive film that reacted with the secondary amine in the CF matrix. Model (**4**) is the ring-opening reaction of the remanent epoxy group in the adhesive film that reacted with the OH group in the CF matrix (etherification). Considering that TFAA can react with OH, NH, and epoxy groups^27–29^, 16 reactive sites existed in the monomers of the CF matrix and the adhesive before the curing reaction. After the curing, on the other hand, 16 and 14 sites can be fluorinated in model (**1**) and models (**2**)-(**4**), respectively, in the monomer structure. Models (**2**)-(**4**) do the number of functional groups to react with TFAA decrease, resulting in the weaker F Kα signal at the interface concerning the surrounding matrix and adhesive.

**Figure 4:**
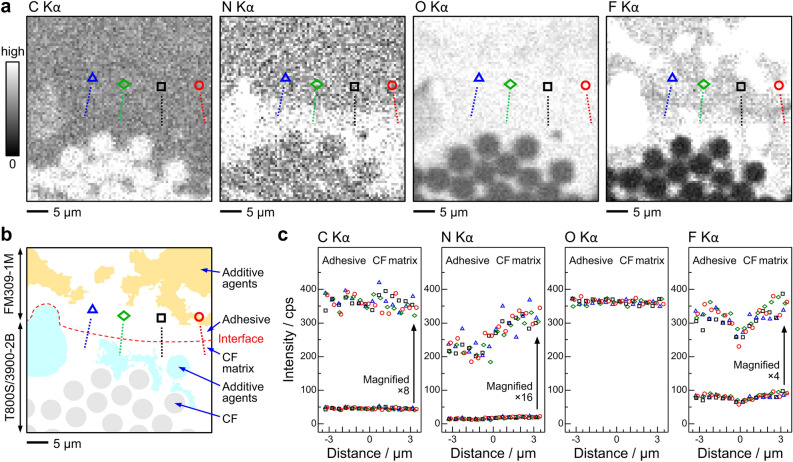
Intensity profile analysis of the soft X-ray images at the CF-matrix/adhesive interface. (**a**) The C, N, O, and F Kα SXM images at the CF-matrix/adhesive interface region. The intensity profile analysis around the interface was performed along the four straight lines with the circle, square, diamond, and triangle symbols. These symbols refer to those used in the intensity plot. (**b**) The segmented image of the CF-matrix/adhesive interface. The intensity profile analysis across the interface was performed along straight lines indicated with the circle, square, diamond, and triangle symbols. (**c**) The intensity profile for the C, N, O, and F Kα emissions at the CF-matrix/adhesive interface. The abscissa is the distance measured from the interface. The circle, square, diamond, and triangle data points correspond to the symbols in the segmented image.

**Figure 5:**
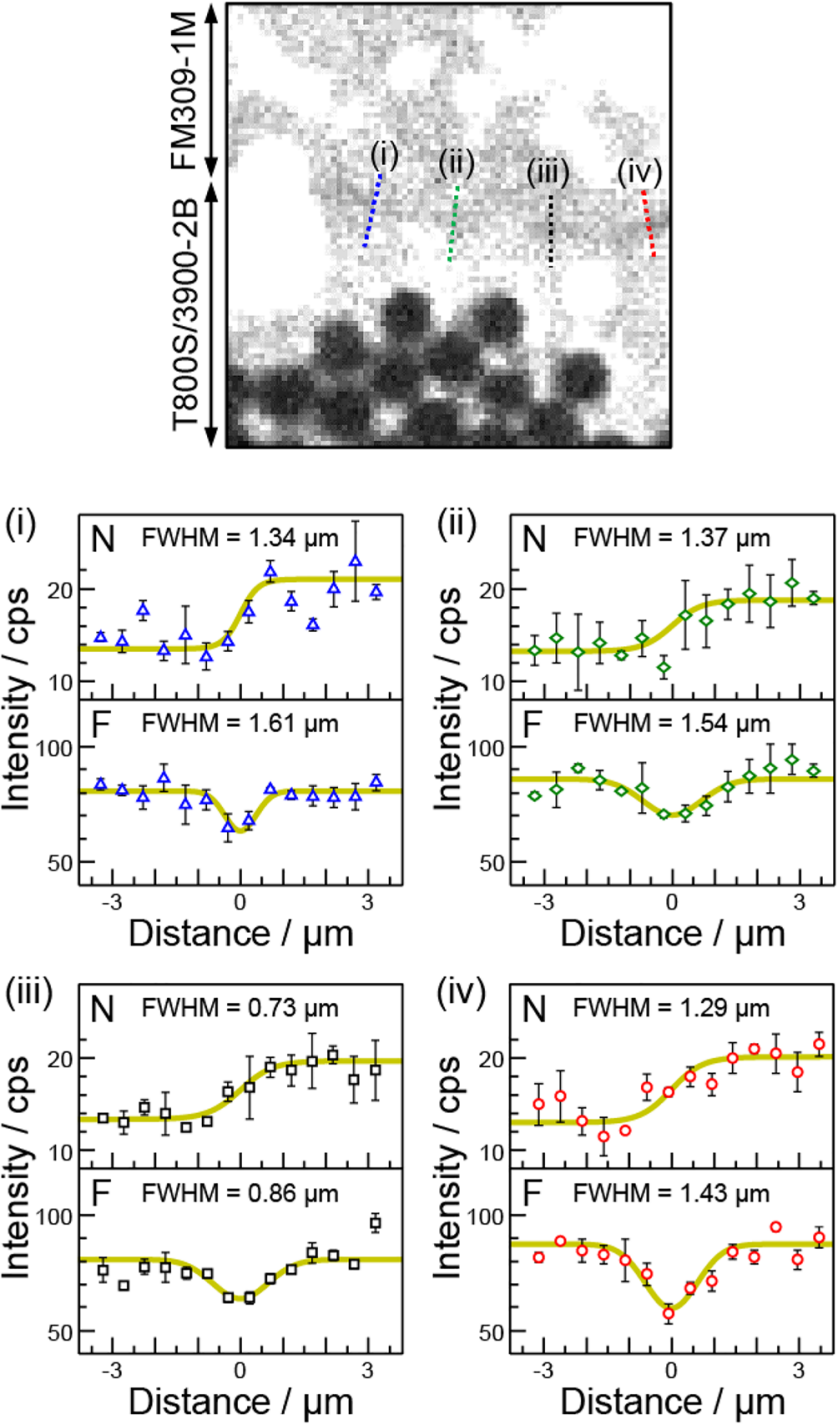
Fitting analysis of the intensity profile in the soft X-ray images. The F Kα image at the CF-matrix/adhesive interface region and the fitting analysis of the N and F Kα intensity profiles at the positions of (i), (ii), (iii), and (iv). The N and F Kα intensity profiles were analyzed using Boltzmann and Gaussian functions, respectively. The best fit results are indicated by the thick solid curve. The FWHM of the best fit curve is provided in each figure. The triangle (i), diamond (ii), square (iii), and circle (iv) data points correspond to the symbols used in Fig. 4.

Figure 4a–c show the intensity profile analysis for the C, N, O, and F Kα images at the CF-matrix/adhesive interface. A change in the intensity profile around the interface was observable for the N and F Kα images, whereas the C and O Kα intensity profiles remained unchanged. The N Kα intensity profile exhibited a step-type feature at the interface, whereas the N Kα intensity from the CF matrix was stronger than that from the adhesive (see Fig. 4c). As discussed above, the observed N Kα contrast was ascribed to the relative amounts of the amine-based curing agents that can react with the epoxy polymers in the CF matrix (TGDDM) and the adhesive (DGEBA). From the intensity profile analysis (Fig. 5), the FWHM of the step-type N Kα intensity profile was 0.7–1.4 μm. On the other hand, the F Kα image exhibited a decrease in F Kα intensity at the interface. As shown in Fig. 4c, the F Kα intensity at the interface decreased by about 20% to that at the bulk epoxy polymers in the adhesive and CF matrix. As shown in Fig. 5, the F Kα intensity profiles were reproduced by the Gaussian function with the FWHM of 0.9–1.6 μm, which was close to the FWHM for the corresponding N Kα intensity profile. From the perspective of the chemical interaction, the decrease in the F Kα intensity at the interface was consistent with models (2)-(4) in Fig. 3b due to covalent bonds between the CF matrix and the adhesive, which has been an open question in epoxy-amine systems^34–38^.

A molecular dynamics (MD) simulation study was recently reported for the cross-linking processes induced by the curing agent and discussed the number of every branched structure type^39^. The DDS curing agent has two primary amine groups and can form one, two, three, or four branches through the cross-linking reaction with epoxy polymers such as TGDDM and DGEBA. The simulations showed that TGDDM-DDS mainly formed three- and four-branched structures through generating the OH group without unreacted molecules^39^. On the other hand, they indicated that one- and two-branched structures were predominant with some epoxy groups in unreacted form for DGEBA-DDS^39^. These results indicate that the OH group in TGDDM-DDS may react in an auto-catalytic manner with the remanent epoxy group in DGEBA-DDS. The present SXM observations can be explained by assuming these etherification reactions.

In conclusion, we used the fluorescence-yield SXM technique to reveal the chemical and physical states at the epoxy-based CF-matrix/adhesive interface for industrial use. We observed evidence for multiple and multiscale bonding factors in the CFRP joining structure, including μm-scale mechanical interlocking and interface-specific chemical states. Notably, we could visualize the distribution of the chemical state peculiar at the CF-matrix/adhesive interface, which exhibited a decrease in F Kα intensity. The observed interface-specific chemical state was consistent with the etherification reactions during the adhesion process. These observations were achieved by the SXM measurement combined with the TFAA treatment, which makes it possible to efficiently visualize the OH group distribution. The experimental proof of the presence of the interface-specific covalent-bond distribution would pave the way for developing reliable adhesive bonding technologies.

## Methods

### Soft X-ray microscopy

The SXM experiment was performed at the soft X-ray undulator beamline BL17SU of SPring-8^40–42^. We used the vertically polarized X-rays with the energy resolving power (*hv*/*ΔE*) of about 9,000. As shown in Fig. 1b, the incident soft X-ray beam was focused with a Fresnel zone plate (FZP), and the first-order diffraction beam was irradiated on the sample through the order sorting aperture (OSA)^43^. The full width at half maximum (FWHM) of the incident X-ray beam (i.e., spatial resolution) was 440 nm. The energy and intensity of the fluorescent X-rays emitted from the sample were analyzed using a silicon drift detector (SDD). After determining the regions of interest in the *μ*-XRF spectra using SDD (Fig. 1c), SXM images were obtained by measuring the *μ*-XRF signals while scanning the sample in the X and Y directions. The angle between the incident X-ray beam axis and the fluorescent X-ray beam axis was 90°, and the incident angle was 25° to the surface normal. The vacuum pressure around the sample was less than 10 Pa.

The *μ*-XAS spectra were obtained by measuring O Kα fluorescent X-rays using SDD as a function of the incident photon energy, and were recorded in the single-point mode. In the present experimental configuration, the elastic (Rayleigh) scattering was also involved in the XAS intensity. We estimated the contribution of the elastic peak in the XAS intensity to be about 17%. We confirmed that the elastic peak contribution had little effect on the XAS spectral shape because the intensity variation of the elastic peak was almost linear at *hv* = 525–555 eV with respect to that of the O Kα signals from the sample.

Irradiation of focused X-rays on polymer materials induces mass loss and subsequent chemical change (see, supplementary information S1 and Ref.^32^). In the present work, to minimize the effect of the irradiation-induced chemical change, the X-ray dose time in the soft X-ray imaging was shorter than that of the critical dose for the R-OH group (see, supplementary information S1).

### Sample preparation

For the SXM experiment, we prepared a joining structure of the unidirectional laminate of the CF prepreg (T800S/3900-2B, Toray Industries, Inc.)^22,23^ bonded by an epoxy-based adhesive film (FM309-1 M, Solvay S.A.)^24^. The CF prepreg (T800S/3900-2B) is constituted of the CF layer and the interlaminar toughened layer in which polyamide particles are dispersed^22^. The stacked CF prepregs were vacuum-packed and introduced to an autoclave for curing. The curing of the CF prepregs was performed at 180 °C for two hours under the molding pressure of 650 kPa. During the curing procedure, the top surface of the CF laminate was protected by a peel ply (1500EV6, Diatex SAS), which was removed after the curing. The adhesive film was then attached to the bare surface of the CFRP laminate to make the CFRP-CFRP joining structure and was heat-treated at 180 °C for two hours under the molding pressure of 300 kPa for curing of the adhesive film. The cross-section of the CFRP-CFRP joining structure shown in Fig. 1a was obtained by mechanical polishing. The cross-section sample was introduced into the TFAA atmosphere at room temperature and atmospheric pressure for one hour. After the TFAA treatment, the sample was exposed to a vacuum to remove the unreacted TFAA. After the TFAA treatment, we did not apply any additional chemical treatment such as post curing; that is, the TFAA treatment does not affect the density of the OH group around the adhesive interface. Before the present work, furthermore, we confirmed the validity of the experimental data of the sample after the TFAA treatment by measuring O 1 s XPS and O K-edge XAS for TGDDM-DDS as shown in the supplementary information S1.

## Supplementary Information

Supplementary Information.

## Data Availability

The data supporting this study's findings is available from the corresponding author upon reasonable request.
