# Supplementary material for: Visualizing interface-specific chemical bonds in adhesive bonding of carbon fiber structural composites using soft X-ray microscopy
Source: Sci Rep. 2022 Sep 29;12:16332. doi: 10.1038/s41598-022-20233-4 (PMC9522830; doi:10.1038/s41598-022-20233-4)
Supplement: Supplementary file 1 — Supplementary Information. [file 41598_2022_20233_MOESM1_ESM.docx]

**Supplementary Information**

**Visualizing interface-specific chemical bonds
in adhesive bonding of carbon fiber structural composites
using soft X-ray microscopy**

Hiroyuki Yamane,^1^ Masaki Oura,^1^ Noriko Yamazaki,^2^ Tomoko Ishihara,^1^ Koichi Hasegawa,^3^

Tetsuya Ishikawa,^1^ Kiyoka Takagi,^4^ and Takaki Hatsui^1^

1. RIKEN SPring-8 Center, RIKEN, Kouto, Sayo, Hyogo 679-5148, Japan.
2. Chemical Research Department, Research & Innovation Center, Mitsubishi Heavy Industries, Ltd., Minato-mirai, Yokohama, Kanagawa 220-8401, Japan.
3. Manufacturing Technology Research Department, Research & Innovation Center, Mitsubishi Heavy Industries, Ltd., Oye, Nagoya, Aichi 455-8515, Japan.
4. Fixed Wing Aircraft Engineering Department, Integrated Defense & Space Systems, Mitsubishi Heavy Industries, Ltd., Toyoyama, Nishikasugai, Aichi 480-0293, Japan.

**Radiation damage in the SXM experiment**


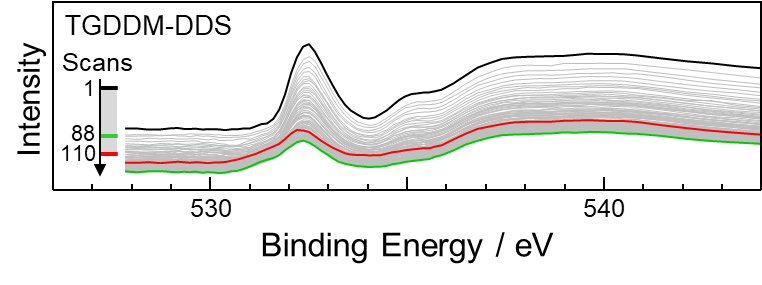


**Figure S1.** O 1s XAS spectra of TGDDM-DDS as a function of the scan cycles.

Figure S1 shows the XAS spectra for TGDDM-DDS as a function of the scan cycles. This measurement was performed using a conventional XAS system with an X-ray beam diameter of about 1 mmφ.^1,2^ As the scan cycle increased, the pre-edge feature at 532.5 eV weakened with a shoulder feature appearing at 531.2 eV. The decrease in the XAS intensity at 532.5 eV exhibited an exponential-like decay due to a mass loss accompanied by photoionization. The shoulder structure at 531.2 eV might originate from the formation of damage-induced chemical species.^2^

To understand the radiation damage of the sample by the focused X-ray beam, we evaluated the absorbed dose (*D*_a_) in the present experiment. As reported in our previous works,^1-3^ we assumed that *D*_a_ was equal to the kinetic energy released in the material (Kerma) under the charged-particle equilibrium. Kerma can be expressed as the product of the irradiated photons and the absorbed energy per unit mass by one photon,

$D_{a}=Kerma=N_{\mathrm{ph}}t_{\mathrm{dose}}\times\frac{E_{p}\left[ 1-T(E) \right]}{\lambda S\rho}$ (Gy),

where *N*_ph_, *t*_dose_, *E*_p_, *T*(*E*), *λ*, *S*, and *ρ* represent the photon flux, the photon dose time, the photon energy, the transmission probability of the materials, the probing depth, the photon beam size, and the density of the materials, respectively.

In the present work, *N*_ph_, *E*_p_, and FWHM were 8×10^8^ photons/s, 750 eV, and 440 nm, respectively. Considering the incident X-ray angle, *S* on the sample surface was 0.17 μm^2^ in the ±σ standard deviation of the Gaussian distribution. If 68.2% of photons existed within ±σ of the x-ray beam, the photon flux density (*N*_ph_/*S*) could be calculated as 3.2×10^15^ photons/mm^2^/s. Considering references 1-4, the X-ray irradiation time that reaches the critical dose for R-OH was estimated to be 1.35 s. Based on this, to minimize the possible mass loss and resultant chemical change upon irradiation, we obtained the X-ray images at the scanning rate of 1 s/pixel.

We note that the microprobe O K-edge XAS spectra might involve the irradiation effect. If irradiation-induced chemical changes occurred, new XAS peaks would appear in the low-*hv* region.^2^ In Fig. 2(e) of the original manuscript, evidence for the new peak formation due to the chemical change was not observable. Therefore, we consider that the present XAS spectra contain the effect of the mass loss, not the subsequent chemical change.

**Effect of TFAA treatment**

The TFAA treatment is described by the following formula. We confirmed the validity of the experimental data by measuring XPS and XAS before and after the TFAA treatment. The confirmed material was TGDDM-DDS, which is the model material for the CF matrix in T800S/3900-2B.

R−OH + (CF_3_CO)_2_O → R−O(C=O)CF_3_ + CF_3_COOH

Figure S2 shows O 1s XPS spectra of TGDDM-DDS before and after the TFAA treatment. Peaks A and B in XPS spectra originated from the S=O and O−H groups, respectively. The peak area ratio B/A was 2.03, which corresponds well with the O−H/S=O ratio in TGDDM-DDS. After the TFAA treatment, a new peak C due to the O−C=O group appeared as explained by the chemical reaction formula. No additional oxygen-containing chemical species were observed upon fluorination.


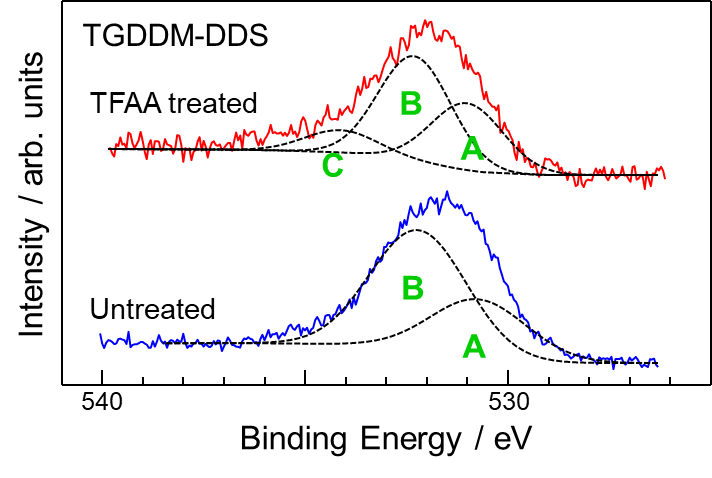


**Figure S2.** O 1s XPS spectra of TGDDM-DDS before and after the TFAA treatment (blue and red curves, respectively). Black dashed curves indicate the convoluted peaks obtained by the least-squares fitting using the Gaussian function. The Shirley method was used for the background subtraction for the peak fitting analysis.

Figure S3 shows O K-edge XAS spectra of TGDDM-DDS before and after the TFAA treatment. The pre-edge peak at 532.5 eV obtained for the untreated TGDDM-DDS was ascribed to the OH···π interaction.^2^ After the TFAA treatment, the pre-edge peak exhibited a low-*hv* shift of 0.1 eV and an increase in FWHM of 0.03 eV due to the formation of the O−C=O group. Other additional features were not observable. These changes upon fluorination do not affect the conclusion of the present work.


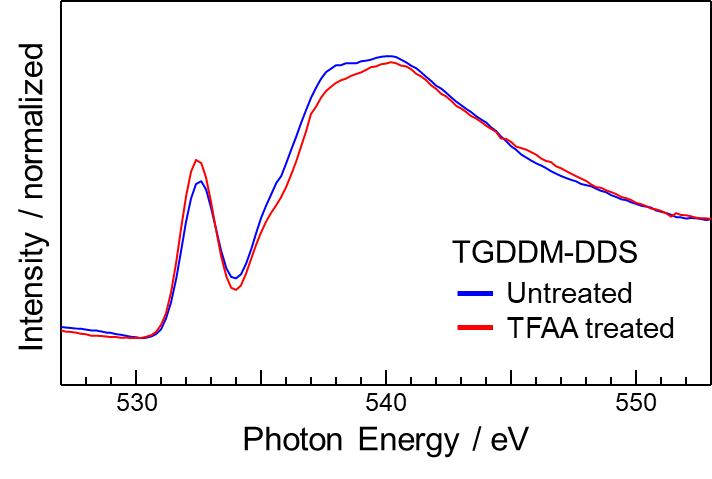


**Figure S3.** O K-edge XAS spectra of TGDDM-DDS before and after the TFAA treatment (blue and red curves, respectively).

**References**

1. Yamane, H., Oura, M., Sawada, K., Ebisu, T., Ishikawa, T., Yamazaki, N., Hasegawa, K., Takagi, K. & Hatsui, T. Critical Absorbed Dose of Resinous Adhesive Material Towards Non-Destructive Chemical-State Analysis Using Soft X-rays, *J. Electron Spectrosc. Relat. Phenom.* **232**, 11–15 (2019).
2. Yamane, H., Oura, M., Takahashi, O., Fons, P., Varadwaj, P. R., Shimoi, Y., Ohkubo, M., Ishikawa, T., Yamazaki, N., Hasegawa, K., Takagi, K. & Hatsui, T. Soft X-ray absorption spectroscopy probes OH···π interactions in epoxy-based polymers. *J. Phys. Chem. C* **120**, 9622–9627 (2020).
3. Yamane, H., Oura, M., Takahashi, O., Ishihara, T., Yamazaki, N., Hasegawa, K., Ishikawa, T., Takagi, K. & Hatsui, T. Physical and chemical imaging of adhesive interfaces with soft X-rays. *Commun. Mater.* **2**, 63 (2021).
4. Wang, J., Morin, C., Li, L., Hitchcock, A. P., Scholl, A. & Doran, A. Radiation damage in soft X-ray microscopy, *J. Electron Spectrosc. Relat. Phenom.* **170**, 25–36 (2009).
